# Supplementary material for: Putative rhythms in attentional switching can be explained by aperiodic temporal structure
Source: Nat Hum Behav. 2022 Jun 9;6(9):1280–91. doi: 10.1038/s41562-022-01364-0 (PMC9489532; doi:10.1038/s41562-022-01364-0)
Supplement: Supplementary file 1 — Supplementary Fig. 1 and Discussion. [file 41562_2022_1364_MOESM1_ESM.pdf]

nature portfolio

https://doi.org/10.1038/s41562-022-01364-0

Supplementary information

Putative rhythms in attentional switching can be explained by aperiodic temporal structure

In the format provided by the authors and unedited

Supplementary information for "Putative rhythms in attentional switching can be explained by aperiodic temporal structure"

Geoffrey Brookshire

Centre for Human Brain Health, University of Birmingham, Birmingham, UK
SPARK Neuro, New York, NY, USA
brookshire@uchicago.edu

Supplementary figures

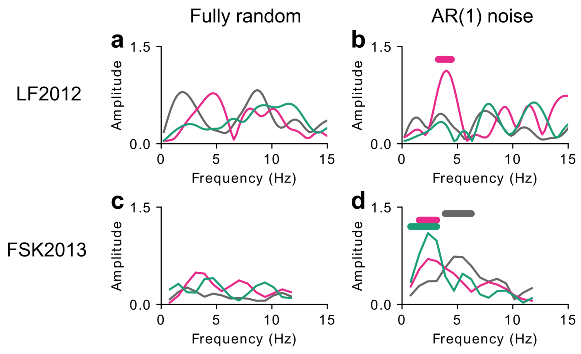

Figure 1: **Examples of false positive rhythms from aperiodic noise.** Each panel illustrates three randomly-selected simulated experiments (shown in different colors). Bars at the top of the panels show frequencies that were identified in each experiment as significant oscillatory components. **(a, c)** Experiments generated with fully random data. **(b, d)** Experiments generated with AR(1) noise that was consistent over trials. **(a, b)** Data analyzed using the procedure from LF2012. **(c, d)** Data analyzed using the procedure from FSK2013.

Supplementary discussion

Neural mechanisms of attentional dynamics

What neural mechanisms influence spontaneous attentional switching? Two major categories of models aim to explain rhythms in behavior via rhythms in brain activity. First, some researchers propose that a 'pacemaker' oscillation determines when attention switches between stimuli: perhaps endogenous oscillations at 3-8 Hz coordinate activity within a thalamo-cortical network, with one phase of each cycle facilitating sensory processing and another phase facilitating shifts in the attentional focus[1,2]. In a second category of

oscillatory models, some researchers propose that attentional switching may depend on competitive inhibition within visual cortex[3]. These competitive dynamics have been suggested to arise due to inter-hemispheric connections[4], or due to center-surround inhibition in overlapping receptive fields[5,6].

The results reported here, however, raise the possibility that attentional switching could show non-oscillatory dynamics. Importantly, non-oscillatory patterns in behavior cannot be accounted for by a pacemaker oscillation or by mutual inhibition between overlapping receptive fields. What neural mechanisms could give rise to aperiodic attentional switching?

Aperiodic patterns of neural activity appear in a range of different behaviors and brain areas. For example, zebra finches sing motifs that are very similar across repetitions, and each repetition is accompanied by a highly stereotyped sequence of neural activity in the song-production area HVC[7]. Furthermore, rats show consistent aperiodic sequences of hippocampal spiking during working memory. These sequences are specific to the item being remembered[8]. Aperiodic patterns such as these may arise due to inherent properties of networks in the brain[9–12], such as recurrent connections and a variety of time-dependent physiological processes (e.g. short-term synaptic plasticity)[13].

Research with artificial neural networks supports the idea that inherent network properties can give rise to aperiodic patterns of activity[10,14]. In particular, sparsely-connected recurrent neural networks (RNNs) capture a number of the characteristics of attentional switching. For example, they show chaotic spontaneous dynamics[15], mirroring the way attention moves even without changes to the stimulus. Sparse RNNs are not limited to chaotic activity. These networks show reproducible dynamic patterns when they are stimulated with the same input[16], analogous to behavioral patterns like the attentional blink. Furthermore, a single RNN can flexibly generate multiple distinct behaviors, with different inputs corresponding to different dynamic responses[17,18]. Correspondingly, different cues lead to different patterns of attentional switching (e.g. cue in the right versus left visual hemifield,[19]).

These computational and experimental findings motivate a hypothesis about the dynamics of attention: Non-oscillatory endogenous attentional switching may arise due to recurrent connections and time-dependent physiological processes within the attentional network. Prior research shows that the attentional focus moves as a function of the constantly-evolving population activity in brain areas such as the frontal eye fields (FEF)[20–22]. Reproducible patterns of aperiodic switching may emerge as a consequence of recurrent connections and time-dependent physiological processes in attentional areas in the brain. If this hypothesis is true, endogenous oscillations may not be necessary to account for patterns in attentional switching.

To discriminate between these oscillatory and non-oscillatory hypotheses, future studies could examine attentional switching in response to a sequence of multiple cue events. If attentional switching depends on ongoing oscillations that are reset by transient events (such as the cue stimulus)[19,23–25], then the time-

course of attention should not depend on whether the cue was preceded by another event. In contrast, recurrent network dynamics are sensitive to the initial conditions at the time that an event occurs[13,14]. As a consequence of this context-dependence, this recurrent-connection hypothesis predicts that the time-course of attention will differ based on the amount of time between the two cue stimuli.

Do the alternative methods specifically identify oscillations?

Given a strong oscillation in behavior, the AR surrogate and robust est. analyses can positively identify that oscillation. These methods identify oscillations with higher selectivity (Figure 4) than the standard approach of shuffling-in-time. The robust est. method has been widely used in climate science for 25 years, helping to identify real oscillations in the presence of autocorrelated noise.

But what can we say about the converse situation: Given a positive statistical result, how confident can we be that an oscillatory process gave rise to that result? In electrophysiological studies, non-rhythmic evoked activity (such as an event-related potential; ERP) can sometimes appear as a brief increase in power at a particular frequency band. Could a similar non-oscillatory process give rise to significant results using the AR surrogate and robust est. methods? In principle, attentional re-orienting could show a pattern analogous to ERPs, in which the cue event is followed by a consistent non-oscillatory response. If that behavioral 'ERP' appears as a sequence of 2 to 3 fairly rhythmic cycles, then the methods presented here would be likely to identify it as an oscillation. The difference between sustained oscillations and brief bursts of band-limited activity has strong implications for the interpretation of these results[26–28]. To distinguish between oscillations and non-oscillatory bursts, future studies could extend the range of the behavioral time-series to test whether any putative oscillations are sustained over time. If future studies uncover significant behavioral oscillations using the AR surrogate or robust est. methods, they will need to design experiments that are capable of disentangling ongoing oscillations from 'bursty' evoked patterns.

Anti-phase relationships in behavior

A number of studies have argued for rhythmic attentional switching on the basis of an anti-phase relationship in the spectra of behavior toward the cued and uncued stimuli[19,23,29–32]. This anti-phase relationship, they suggest, indicates that attention switches between locations at the frequency of the spectral peak. Although an anti-phase relationship does suggest that attention switches between stimuli, it does not necessarily reflect rhythmic switching. For example, inverting a white-noise time-course leads to an anti-phase relationship without any oscillations. Anti-phase dynamics are further evidence of rich temporal dynamics in attention, but they do not uniquely point to periodic rhythms in behavior.

Characterizing aperiodic structure in behavior

How can we measure and conceptualize the aperiodic structure in behavior? In both the AR surrogate and robust est. methods, aperiodic structure is measured by estimating the AR(1) coefficient that best fits the data. This AR(1) coefficient describes how strongly the signal correlates with itself across time, and therefore provides a measure of how quickly and consistently attention switches locations. Both of these methods could also be adapted to examine 'power-law' structure in behavior by fitting the data to a $1/f^\beta$ spectrum instead of to an AR(1) process. This power-law approach has been widely applied to behavior on the scale of minutes to hours[33–35], but not yet on the scale of milliseconds to seconds. Furthermore, future research on attentional switching could examine concrete, non-repeating features of the behavioral time-series. This approach could identify features analogous to the attentional blink or inhibition of return, clarifying how attention dynamically samples the perceptual scene after a cuing event. An aperiodic view of temporal structure will enrich our understanding of attention and the neural processes that support it.

Related randomization measures

Neuroscience studies often determine statistical significance using randomization tests. Shuffling-in-time and the AR surrogate method were both developed within this wider context of using the data to infer the distribution under the null hypothesis. Cluster-based permutation tests, for example, are widely used to test for differences between experimental conditions in electrophysiological data[36,37]. In addition, some studies use "random shifting in time" to test for a consistent phase relationship between two signals[38–41]. In this method, the surrogate distribution is derived by re-calculating the phase relationship after randomly shifting one of the signals in time. Random shifting preserves the spectral and temporal characteristics of each of the two signals, but destroys any relationship between them. Surrogate tests provide a powerful statistical tool that must be carefully tuned to the hypothesis in question.

References

[1] Ian C Fiebelkorn and Sabine Kastner. A rhythmic theory of attention. *Trends in Cognitive Sciences*, 23(2):87–101, 2019.

[2] Randolph F Helfrich, Assaf Breska, and Robert T Knight. Neural entrainment and network resonance in support of top-down guided attention. *Current Opinion in Psychology*, 29:82–89, 2019.

[3] Ayelet N Landau. Neuroscience: A mechanism for rhythmic sampling in vision. *Current Biology*, 28(15):R830–R832, 2018.

[4] Ayelet N Landau, Helene M Schreyer, Stan Van Pelt, and Pascal Fries. Distributed attention is implemented through theta-rhythmic gamma modulation. *Current Biology*, 25(17):2332–2337, 2015.

[5] Ricardo Kienitz, Joscha T Schmiedt, Katharine A Shapcott, Kleopatra Kouroupaki, Richard C Saunders, and Michael Christoph Schmid. Theta rhythmic neuronal activity and reaction times arising from cortical receptive field interactions during distributed attention. *Current Biology*, 28(15):2377–2387, 2018.

[6] Samson Chota, Canhuang Luo, Sébastien M Crouzet, Léa Boyer, Ricardo Kienitz, Michael Christoph Schmid, and Rufin VanRullen. Rhythmic fluctuations of saccadic reaction time arising from visual competition. *Scientific Reports*, 8(1):1–7, 2018.

[7] Richard HR Hahnloser, Alexay A Kozhevnikov, and Michale S Fee. An ultra-sparse code underliesthe generation of neural sequences in a songbird. *Nature*, 419(6902):65–70, 2002.

[8] Eva Pastalkova, Vladimir Itskov, Asohan Amarasingham, and György Buzsáki. Internally generated cell assembly sequences in the rat hippocampus. *Science*, 321(5894):1322–1327, 2008.

[9] Dean V Buonomano and Michael M Merzenich. Temporal information transformed into a spatial code by a neural network with realistic properties. *Science*, 267(5200):1028–1030, 1995.

[10] Dean V Buonomano and Wolfgang Maass. State-dependent computations: spatiotemporal processing in cortical networks. *Nature Reviews Neuroscience*, 10(2):113–125, 2009.

[11] Dezhe Z Jin, Naotaka Fujii, and Ann M Graybiel. Neural representation of time in cortico-basal ganglia circuits. *Proceedings of the National Academy of Sciences*, 106(45):19156–19161, 2009.

[12] Vladimir Itskov, Carina Curto, Eva Pastalkova, and György Buzsáki. Cell assembly sequences arising from spike threshold adaptation keep track of time in the hippocampus. *Journal of Neuroscience*, 31(8):2828–2834, 2011.

[13] Dean V Buonomano. Neural dynamics based timing in the subsecond to seconds range. In Hugo Merchant and Victor de Lafuente, editors, *Neurobiology of Interval Timing*, pages 101–117. Springer, New York, 2014.

[14] Saurabh Vyas, Matthew D Golub, David Sussillo, and Krishna V Shenoy. Computation through neural population dynamics. *Annual Review of Neuroscience*, 43:249–275, 2020.

[15] Haim Sompolinsky, Andrea Crisanti, and Hans-Jurgen Sommers. Chaos in random neural networks. *Physical Review Letters*, 61(3):259, 1988.

[16] Uma R Karmarkar and Dean V Buonomano. Timing in the absence of clocks: encoding time in neural network states. *Neuron*, 53(3):427–438, 2007.

[17] Jian K Liu and Dean V Buonomano. Embedding multiple trajectories in simulated recurrent neural networks in a self-organizing manner. *Journal of Neuroscience*, 29(42):13172–13181, 2009.

[18] David Sussillo and Larry F Abbott. Generating coherent patterns of activity from chaotic neural networks. *Neuron*, 63(4):544–557, 2009.

[19] Ayelet N Landau and Pascal Fries. Attention samples stimuli rhythmically. *Current Biology*, 22(11):1000–1004, 2012.

[20] Tirin Moore and Mazyar Fallah. Microstimulation of the frontal eye field and its effects on covert spatial attention. *Journal of Neurophysiology*, 91(1):152–162, 2004.

[21] Timothy J Buschman and Earl K Miller. Serial, covert shifts of attention during visual search are reflected by the frontal eye fields and correlated with population oscillations. *Neuron*, 63(3):386–396, 2009.

[22] Corentin Gaillard, Sameh Ben Hadj Hassen, Fabio Di Bello, Yann Bihan-Poudec, Rufin VanRullen, and Suliann Ben Hamed. Prefrontal attentional saccades explore space rhythmically. *Nature Communications*, 11(1):1–13, 2020.

[23] Ian C Fiebelkorn, Yuri B Saalmann, and Sabine Kastner. Rhythmic sampling within and between objects despite sustained attention at a cued location. *Current Biology*, 23(24):2553–2558, 2013.

[24] Peter Lakatos, Joachim Gross, and Gregor Thut. A new unifying account of the roles of neuronal entrainment. *Current Biology*, 29(18):R890–R905, 2019.

[25] Jonas Obleser and Christoph Kayser. Neural entrainment and attentional selection in the listening brain. *Trends in cognitive sciences*, 23(11):913–926, 2019.

[26] Joseph Feingold, Daniel J Gibson, Brian DePasquale, and Ann M Graybiel. Bursts of beta oscillation differentiate postperformance activity in the striatum and motor cortex of monkeys performing movement tasks. *Proceedings of the National Academy of Sciences*, 112(44):13687–13692, 2015.

[27] Stephanie R Jones. When brain rhythms aren't 'rhythmic': implication for their mechanisms and meaning. *Current Opinion in Neurobiology*, 40:72–80, 2016.

[28] Freek van Ede, Andrew J Quinn, Mark W Woolrich, and Anna C Nobre. Neural oscillations: sustained rhythms or transient burst-events? *Trends in Neurosciences*, 41(7):415–417, 2018.

[29] Kun Song, Ming Meng, Lin Chen, Ke Zhou, and Huan Luo. Behavioral oscillations in attention: rhythmic α pulses mediated through θ band. *Journal of Neuroscience*, 34(14):4837–4844, 2014.

[30] Yan Huang, Lin Chen, and Huan Luo. Behavioral oscillation in priming: competing perceptual predictions conveyed in alternating theta-band rhythms. *Journal of Neuroscience*, 35(6):2830–2837, 2015.

[31] Laura Dugué, Mariel Roberts, and Marisa Carrasco. Attention reorients periodically. *Current Biology*, 26(12):1595–1601, 2016.

[32] Hao Tam Ho, Johahn Leung, David C Burr, David Alais, and Maria Concetta Morrone. Auditory sensitivity and decision criteria oscillate at different frequencies separately for the two ears. *Current Biology*, 27(23):3643–3649, 2017.

[33] Thomas L Thornton and David L Gilden. Provenance of correlations in psychological data. *Psychonomic Bulletin & Review*, 12(3):409–441, 2005.

[34] Christopher T Kello, Gordon DA Brown, Ramon Ferrer-i Cancho, John G Holden, Klaus Linkenkaer-Hansen, Theo Rhodes, and Guy C Van Orden. Scaling laws in cognitive sciences. *Trends in Cognitive Sciences*, 14(5):223–232, 2010.

[35] J Matias Palva, Alexander Zhigalov, Jonni Hirvonen, Onerva Korhonen, Klaus Linkenkaer-Hansen, and Satu Palva. Neuronal long-range temporal correlations and avalanche dynamics are correlated with behavioral scaling laws. *Proceedings of the National Academy of Sciences*, 110(9):3585–3590, 2013.

[36] Eric Maris and Robert Oostenveld. Nonparametric statistical testing of EEG- and MEG-data. *Journal of Neuroscience Methods*, 164(1):177–190, 2007.

[37] Jona Sassenhagen and Dejan Draschkow. Cluster-based permutation tests of meg/eeg data do not establish significance of effect latency or location. *Psychophysiology*, 56(6):e13335, 2019.

[38] Ryan T Canolty, Erik Edwards, Sarang S Dalal, Maryam Soltani, Srikantan S Nagarajan, Heidi E Kirsch, Mitchel S Berger, Nicholas M Barbaro, and Robert T Knight. High gamma power is phase-locked to theta oscillations in human neocortex. *Science*, 313(5793):1626–1628, 2006.

[39] Bradley Voytek, Mark D'Esposito, Nathan Crone, and Robert T Knight. A method for event-related phase/amplitude coupling. *NeuroImage*, 64:416–424, 2013.

[40] Geoffrey Brookshire, Jenny Lu, Howard C Nusbaum, Susan Goldin-Meadow, and Daniel Casasanto. Visual cortex entrains to sign language. *Proceedings of the National Academy of Sciences*, 114(24):6352–6357, 2017.

[41] Anne Keitel, Robin AA Ince, Joachim Gross, and Christoph Kayser. Auditory cortical delta-entrainment interacts with oscillatory power in multiple fronto-parietal networks. *NeuroImage*, 147:32–42, 2017.
