# Supplementary material for: Putative rhythms in attentional switching can be explained by aperiodic temporal structure
Source: Nat Hum Behav. 2022 Jun 9;6(9):1280–91. doi: 10.1038/s41562-022-01364-0 (PMC9489532; doi:10.1038/s41562-022-01364-0)
Supplement: Supplementary file 2 — Reporting Summary [file 41562_2022_1364_MOESM2_ESM.pdf]

nature portfolio

Corresponding author(s): Geoffrey Brookshire

Last updated by author(s): Apr 23, 2022

Reporting Summary

Nature Portfolio wishes to improve the reproducibility of the work that we publish. This form provides structure for consistency and transparency in reporting. For further information on Nature Portfolio policies, see our Editorial Policies and the Editorial Policy Checklist.

Statistics

For all statistical analyses, confirm that the following items are present in the figure legend, table legend, main text, or Methods section.

n/a	Confirmed	
☐	☒	The exact sample size (*n*) for each experimental group/condition, given as a discrete number and unit of measurement
☐	☒	A statement on whether measurements were taken from distinct samples or whether the same sample was measured repeatedly
☐	☒	The statistical test(s) used AND whether they are one- or two-sided<br>*Only common tests should be described solely by name; describe more complex techniques in the Methods section.*
☐	☒	A description of all covariates tested
☐	☒	A description of any assumptions or corrections, such as tests of normality and adjustment for multiple comparisons
☐	☒	A full description of the statistical parameters including central tendency (e.g. means) or other basic estimates (e.g. regression coefficient) AND variation (e.g. standard deviation) or associated estimates of uncertainty (e.g. confidence intervals)
☐	☒	For null hypothesis testing, the test statistic (e.g. *F*, *t*, *r*) with confidence intervals, effect sizes, degrees of freedom and *P* value noted<br>*Give P values as exact values whenever suitable.*
☒	☐	For Bayesian analysis, information on the choice of priors and Markov chain Monte Carlo settings
☐	☒	For hierarchical and complex designs, identification of the appropriate level for tests and full reporting of outcomes
☐	☒	Estimates of effect sizes (e.g. Cohen's *d*, Pearson's *r*), indicating how they were calculated

Our web collection on statistics for biologists contains articles on many of the points above.

Software and code

Policy information about availability of computer code

Data collection	No new data-sets were collected for this study. Simulated data were generated using the code described below under "Data analysis."
Data analysis	All code used to perform these analyses is available online at https://github.com/gbrookshire/simulated_rhythmic_sampling. These analyses were performed using open-source Python code. The exact versions of all external libraries are specified in "requirements.txt" at the Github repository.

For manuscripts utilizing custom algorithms or software that are central to the research but not yet described in published literature, software must be made available to editors and reviewers. We strongly encourage code deposition in a community repository (e.g. GitHub). See the Nature Portfolio guidelines for submitting code & software for further information.

Data

Policy information about availability of data

All manuscripts must include a data availability statement. This statement should provide the following information, where applicable:

- Accession codes, unique identifiers, or web links for publicly available datasets
- A description of any restrictions on data availability
- For clinical datasets or third party data, please ensure that the statement adheres to our policy

All simulated data is available at a public repository: https://osf.io/6bs4e/. The paper provides links to all previously-published datasets that were reanalyzed here.

Field-specific reporting

Please select the one below that is the best fit for your research. If you are not sure, read the appropriate sections before making your selection.

☐ Life sciences ☒ Behavioural & social sciences ☐ Ecological, evolutionary & environmental sciences

For a reference copy of the document with all sections, see nature.com/documents/nr-reporting-summary-flat.pdf

Behavioural & social sciences study design

All studies must disclose on these points even when the disclosure is negative.

Study description

Quantitative study based on computational simulations and reanalysis of publicly-available data.

Research sample

We re-analyzed data from four papers.
- Ho et al. (2017): "Twenty healthy adults (7 male, 3 left-handed, mean age 21.8 ± 3.9) with normal hearing" from around the University of Sydney.
- Davidson et al. (2018): "34 healthy individuals (21 females, 1 left-handed, average age 23 ± 4.7) were recruited via convenience sampling at Monash University, Melbourne, Australia".
- Senoussi et al. (2019): "Thirteen human observers (nine women, four men; age [M ± SD] = 20.9 ± 0.8 years; range: 20–22)" around Paris Descartes University.
- Michel et al. (2021): "Fourteen participants participated in the main study (10 women, 13 right-handed, 11 right-eye dominant, aged 18–28 years, Mage = 21.4, SDage = 2.6)." Participants were recruited from the University of Muenster.

Sampling strategy

Reanalyzed data-sets used convenience samples, with sample sizes based on previous research in the literature.

Data collection

Reanalyzed data-sets were collected on computers. The experimental conditions were manipulated on a trial-by-trial basis, and by virtue of the design any experimenter would have been blind to the relevant experimental condition (i.e. the delay between the cue and the target stimulus).

Timing

The reanalyzed data-sets do not specify the dates of data collection.

Data exclusions

I reanalyzed the data as provided by the original study authors. No additional data were excluded from the analyses.
- Ho et al. (2017): None specified.
- Davidson et al. (2018): None specified.
- Senoussi et al. (2019): "Due to technical issues during data recording, two observers were excluded from the analysis."
- Michel et al. (2021): "An additional participant did not complete the preregistered minimum number of sessions and was therefore excluded. One participant had previously participated in the pilot experiment."

Non-participation

- Ho et al. (2017): None specified.
- Davidson et al. (2018): None specified.
- Senoussi et al. (2019): None specified.
- Michel et al. (2021): "An additional participant did not complete the preregistered minimum number of sessions and was therefore excluded."

Randomization

The experimental conditions were varied within-subjects on a trial-by-trial basis.

Reporting for specific materials, systems and methods

We require information from authors about some types of materials, experimental systems and methods used in many studies. Here, indicate whether each material, system or method listed is relevant to your study. If you are not sure if a list item applies to your research, read the appropriate section before selecting a response.

Materials & experimental systems

n/a	Involved in the study	
☒	☐	Antibodies
☒	☐	Eukaryotic cell lines
☒	☐	Palaeontology and archaeology
☒	☐	Animals and other organisms
☐	☒	Human research participants
☒	☐	Clinical data
☒	☐	Dual use research of concern

Methods

n/a	Involved in the study	
☒	☐	ChIP-seq
☒	☐	Flow cytometry
☒	☐	MRI-based neuroimaging

Human research participants

Policy information about studies involving human research participants

Population characteristics	See above.
Recruitment	Convenience samples. All manipulations were performed within-subjects, so it is not likely that biases in recruiting substantially changed the results.
Ethics oversight	- Ho et al. (2017): "The study was approved by the Human Research Ethics Committees of the University of Sydney."<br>- Davidson et al. (2018): "Monash University Human Research and Ethics Committee approved this study"<br>- Senoussi et al. (2019): "All procedures were approved by the CERES (Conseil d'Évaluation Éthique pour les Recherches En Santé) ethics committee of Paris Descartes University."<br>- Michel et al. (2021): "approved by the ethics committee of the faculty of psychology and sports science, University of Muenster (#2018-36-RM)"

Note that full information on the approval of the study protocol must also be provided in the manuscript.
